# Identification of Depression and Screening for Work Disabilities among Long-Term Unemployed People

**DOI:** 10.3390/ijerph15050909

**Published:** 2018-05-03

**Authors:** Kirsti Nurmela, Aino Mattila, Virpi Heikkinen, Jukka Uitti, Aarne Ylinen, Pekka Virtanen

**Affiliations:** 1 Faculty of Social Sciences, Health Sciences, University of Tampere, 33014 Tampere, Finland; aino.mattila@pshp.fi (A.M.); pekka.j.virtanen@staff.uta.fi (P.V.); 2 Mental Health and Substance Abuse Services, 33900 Tampere, Finland; 3 Department of Adult Psychiatry, Tampere University Hospital, 33521 Tampere, Finland; 4 Department of Neurosciences and Rehabilitation, Tampere University Hospital, 33521 Tampere, Finland; virpi.heikkinen@pshp.fi; 5 Faculty of Medicine and Life Sciences, University of Tampere, 33521 Tampere, Finland; jukka.uitti@staff.uta.fi; 6 Clinic of Occupational Medicine, Tampere University Hospital, 33521 Tampere, Finland; 7 Finnish Institute of Occupational Health, 33100 Tampere, Finland; 8 Department of Neurological Sciences, Faculty of Medicine, University of Helsinki, 00100 Helsinki, Finland; aarne.ylinen@hus.fi; 9 Department of Neurology, Helsinki University Central Hospital, 00260 Helsinki, Finland; 10 Department of Public Health, Faculty of Medicine, Uppsala University, 75124 Uppsala, Sweden

**Keywords:** ability to work, depression, disability pension, health care, identification, unemployment

## Abstract

The study explores whether clinical screening targeted at work disabilities among long-term unemployed people reveals eligible individuals for a disability pension and the importance of depression in granting the disability pensions. A total of 364 participants of the screening project were considered as eligible to apply for disability pension. Among them, 188 were diagnosed as clinically depressed. They were classified into those with earlier depression diagnosis (*n* = 85), those whose depression had not been diagnosed earlier (*n* = 103), and those without diagnosed depression (*n* = 176). The association of this ‘Depression identification pattern’ with being granted a disability pension was explored by logistic regression analyses. Compared to those with earlier diagnosis, those whose depression had not been diagnosed earlier were granted disability pension more commonly (72% vs. 54% OR 2.2, *p* = 0.012). Corresponding figures of the undepressed were 73%, OR 2.3, *p* = 0.002. The adjustments did not affect the results. Clinical examination of the long-term unemployed people in terms of work disability seems to be worthwhile. In particular, the examination reveals new depression diagnoses, which contribute more to the award of disability pension than depression diagnosed earlier by regular health care. Novel ways to detect depression among the unemployed should be implemented in the health and employment services.

## 1. Introduction

Unemployment is the one of the most crucial social determinants of health inequalities. It is associated with increased morbidity and mortality alongside various accompanying financial and social problems [1,2,3,4,5,6]. The interaction between unemployment and ill-health is accounted for through both selection and causation mechanisms [5,7,8]. Connections between unemployment and certain health problems, such as musculoskeletal and mental health disorders, seem to be eminently obvious [9,10,11]. Among mental health problems, depression is closely related to unemployment [7,11,12,13,14,15]. Job loss predicts depression, which impairs the capacity for work and hence increases the risk of drifting towards prolonged unemployment [11,16]. 

Mediated by economic difficulties, unemployment impairs self-esteem, increases mental strain, the risk of poor living conditions and limited opportunities for health care services and, especially concerning long-term unemployment, also relates to impaired ability to work. Previous studies have revealed that long-term unemployed people had from three to four-times higher risk for impaired working ability than those without experiences of unemployment [17]. In earlier research, self-rated poor future working capacity, depression, prolonged unemployment, low level of education, economic difficulties, and higher age were attributes associated with poor working ability among unemployed people [17,18,19,20]. 

However, the assessment of one’s working ability is frequently a complicated and time-consuming process, especially among those people with protracted unemployment periods who have become alienated from the labor market and job-related health requirements [20,21]. Moreover, the symptoms of depression may also complicate the work capacity evaluation, since clinicians may classify them as natural consequences of prolonged unemployment that jobless people should cope with. One reason for the complexity in the assessment of work ability may be the peculiar patterns of the utilization of health services among unemployed people. The symptoms of depression such as hopelessness, fatigue, and lowered self-esteem typically inhibit employability and motivation to seek treatment and rehabilitation [22]. According to earlier findings, diminished social support and social pressure as well as economic strain inhibit the motivation of long-term unemployed people to seek treatment [23]. Furthermore, unmet care needs have been revealed among unemployed people and among people with mental health disorders [23,24,25].

Considering the extensive research on a connection between impaired health and unemployment, research on work disabilities among long-term unemployed people, particularly in relation to depression, is still scarce. The first steps towards an appropriate treatment and assessment of work ability are identification and diagnosing the depression. Therefore, the aim of this study was to explore to what extent a project targeted at assessing the work ability of long-term unemployed people was able to detect undiagnosed depression, and if depression was associated with permanent work disability among long-term unemployed people, as seen in their having been granted a disability pension. 

## 2. Materials and Methods

The study is based on a nation-level screening project among long-term unemployed clients of employment authorities initiated by The Ministry of Labour in Finland. The aim of the project, called ‘Eligibility for a Disability Pension’ (EDIPE), was to enhance the assessment of work disabilities among long-term unemployed people, to identify those who might actually be unable to work, to offer them a thorough medical examination and to issue a medical certificate to support their applications for a disability pension [26]. The employment authorities referred to the EDIPE project those long-term unemployed people, whose unemployment period had continued over a year, whose employability was limited and who had presented a medical certificate concerning the medical disorders affecting their employability. 

Material for this study was drawn from the medical records of individuals involved in the EDIPE examinations during the period 2001–2006 in a Finnish, middle sized industrial town (Tampere). As background information, copies of their medical history documents were ordered comprehensively from primary and specialized health care. The clinical examinations were performed by a group of specialized physicians. Experienced psychiatrists assessed psychiatric diagnoses, which adhered to the criteria of ICD-10 [27]. A detailed description of the protocol of the EDIPE project and the data collection has been published earlier [28,29].

The research data comprised the medical records of 364 long-term unemployed people who had applied for a disability pension, temporary or permanent, and whose medical history documents were available for at least a three-year period prior to their joining the EDIPE project. Within the EDIPE project only one disability pension application was made. All EDIPE clients whose records included the information on granting or rejecting the applied disability pension were included in the sample. The research was authorized by the registrar of the EDIPE project and approved by the ethics committee of Pirkanmaa Hospital District (ETL-code R06032).

The main explanatory variable of the study ‘Depression identification pattern’, was categorized as: (1). ‘Depression diagnosis in EDIPE and in health care (HC)’ included those diagnosed with depression both in the clinical examination of the EDIPE project and earlier in health care; (2). ‘Depression diagnosis in EDIPE’ included those diagnosed with depression in the examinations of the EDIPE project but not earlier in health care. Thus, this group was composed of those whose depression had remained undiagnosed in health care, independent on duration of the depression and number, if any, visits to health care; (3). ‘No depression’ included clients who were not diagnosed with depression in EDIPE examinations.

The outcome of the study was the granting of a disability pension (yes/no). In Finland, adults who are unable to work for medical reasons and whose ability to work cannot be restored by treatment or rehabilitation procedures may be granted a disability pension [30]. There were no unfinished disability pension application processes when the data collection started. Authorized pension providers made the decisions of the disability pensions.

The socio-demographic and other background variables were classified as follows: Age and the duration of unemployment were categorized into four classes in the bivariate analyses but set as a discrete variable in the multivariate analyses. Marital status was dichotomized to single, including unmarried, divorced or widowed, and married or cohabiting. Occupational status was trichotomized to non-manual workers, including entrepreneurs, unskilled, and skilled manual workers. Duration of unemployment refers to the continuous length of time a person being recorded as a jobseeker before the EDIPE examinations. The variable describing the use of health care services was determined as the number of visits to physicians in primary and specialized health care and hospitalizations during the three years prior to the EDIPE examinations. This was categorized into quartiles in the bivariate analyses and in the multivariate analyses handled as a discrete variable. The number of chronic somatic diseases was trichotomized in the bivariate analyses and in the multivariate analyses was handled as a discrete variable. The variable ‘Alcohol use disorder (AUD) on EDIPE’ consists mostly of alcohol abuse or alcohol dependence (F10.1, F10.2) diagnosed in the EDIPE examinations according to ICD-10 classification [27].

The relations between background variables and ‘Depression identification pattern’ were explored by bivariate analyses using Pearson’s chi-square and Fisher’s exact tests. The association of ‘Depression identification pattern’ with the granting of a disability pension was analyzed with binomial logistic regression, adjusting for various sets of the background variables. The main study question was if the two depression identification patterns differ with respect to the granting of a disability pension. Therefore, we chose the ‘Depression diagnosis in EDIPE and in HC’ group as the reference category of the explanatory variable. The results were expressed by odds ratios (OR) and their corresponding 95% confidence intervals (95% CI). Statistical significance was determined with *p*-value < 0.05. Statistical analyses were carried out with SPSS/Win software version 23, IBM^R^ SPSS^R^ Statistics.

## 3. Results

Among the total 364 individuals, men accounted for 63%, the mean age was 53 years (SD 6.6 years, range 24–63 years), 73% were single and 84% were manual workers, skilled or unskilled. Unemployment had lasted on average 11 years (SD 5.5 years, at maximum 35 years). There were, on average, 11 visits (SD 10.4, range 0–72) to health care in the three years prior to EDIPE examinations, nine percent having no visits at all and 24% having fewer than four visits. Almost everybody (97%) had at least one diagnosis of somatic disease. Diagnoses of AUD were set for 48% (Table 1).

In the EDIPE examinations, depression was diagnosed in 188 (52%) of the participants, and in 85 (45%) of them the depression had been previously diagnosed in health care. Depression was more common in women (58%) than in men (48%) in the EDIPE examinations, and the previously diagnosed depression was clearly more prevalent in women (31%) than in men (19%). In addition to gender, depression diagnosis in health care was statistically significantly associated with short duration of unemployment (maximum six years), and high number of visits (upper quartile) to medical professionals, during the preceding three years (Table 1). 

**Figure 1:**
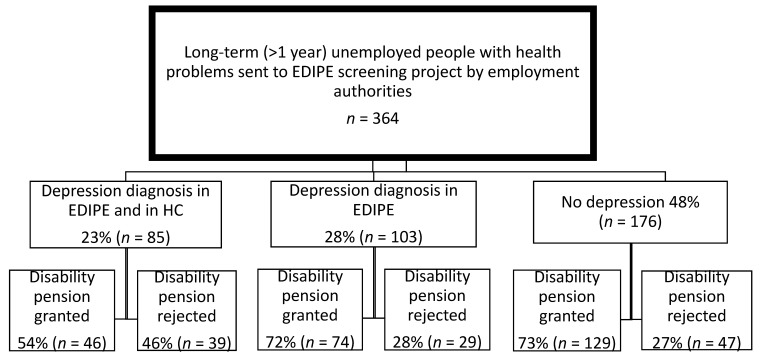
Flow chart of the participants of the EPIDE (Eligibility for a Disability Pension) project according to diagnosed depression and in relation to granting of disability pensions.

Flows of the study sample into disability pensioners in relation to ‘Depression identification pattern’ are presented in Figure 1. The application for a disability pension was accepted in about half (54%) in the ’Depression diagnosis in EDIPE and in HC’ group, while the acceptance rates of ‘Depression diagnosis in EDIPE’ and ‘No depression’ groups were 72% and 73% respectively.

In the logistic regression analysis according to the ‘Depression identification pattern’ (Table 2), the probability for award of a disability pension in the ‘Depression diagnosis in EDIPE’ group was significantly higher (OR 2.16 *p* = 0.012) than in the ‘Depression diagnosis in EDIPE and in HC’ group. The difference remained statistically significant after controlling for sociodemographic background factors, number of visits to health care, and somatic diagnoses. Also, people in the ‘No depression’ group were more likely to be granted disability pensions than those in the ‘Depression diagnosis in EDIPE and in HC’ group.

## 4. Discussion

The present study showed that among the long-term unemployed people believed by the employment authorities to have impaired working capacity, referred to thorough medical examinations on a particular project, and assessed as unable to work, over two thirds were eligible for a disability pension. Depression proved to be the major health problem: It was diagnosed in over half of the total study group and in over half of them the depression had not been diagnosed earlier. Moreover, earlier undiagnosed depression was associated with a significantly greater likelihood of disability pensions being granted than was depression diagnosed in health care prior to the project, independent of somatic comorbidity, number of health care visits, and six additional background factors.

The finding contrasts the reasoning that depression not diagnosed until the EDIPE examinations would be less severe—or less chronic—than depression not diagnosed earlier in health care. Assuming that our outcome, namely disability pension, indicates more severe depression, it is evident that in a considerable percentage of long-term unemployed people suffering from considerable depression, the depression goes undetected by the regular health service system. As the vast majority of our subjects had used health services in the three preceding years, there had been chances for the symptoms of depression to be identified. On the other hand, the visits may have taken place is such a clinical and institutional context that it is not easy to establish the client-physician relationship required for adequate diagnosing—and treatment—of the underlying depression. In addition, the fewer visits, the less likely was the opportunity to diagnose: The proportion of those with very few visits (0–3 in three years) was higher (30% vs. 19%) in the ‘Depression diagnosis in EDIPE’ group than in ‘Depression diagnosis in EDIPE and in HC’ group. The symptoms of depression may also cause people to avoid the health services. However, in this study, adjusting for number of visits to health care did not affect the likelihood of disability pensions being granted. A potential explanation for the result may be that in those cases when depression was identified in health care as the crucial reason for work disability, the disability pension had already been applied for and granted. Therefore, this population with depression as the most essential reason for being granted a disability pension had never even been directed to the EDIPE examinations. In this respect, the assessment of work ability in health care would seem to operate fairly well. Another explanation might be that among the group ‘Depression diagnosis in EDIPE and in HC’ the depression was milder as a result of the effective treatment. Verifying these explanations, however, would require further study and a different research design.

Although the sample is unique, the results of our study are in line with those of earlier studies, showing that the use of health services is reduced during unemployment [31]. Even if unemployed people report their health to be poor more commonly than do employed people [25] and if the unemployed suffer frequently from anxiety and affective disorders [32], they have relatively few physician contacts. The results also corroborate what is already known about the prevalence of depression, its identification, and connection to the granting of disability pensions among long-term unemployed people with disabilities [17]. In particular, this study adds to earlier knowledge about mental health and work ability among the participants of targeted projects. In the group of long-term unemployed people referred to a psychosocial coaching program, over 60% received no disorder-specific treatment [33], and nearly one fifth of long-term unemployed men who participated in a work reintegration program were diagnosed with major depression, yet none of them received treatment for their depression [34]. In a Finnish study of unemployed people, particularly mental health disorders, impaired working capacity seemed to go unnoticed in health care [21].

A peripheral labor market position, such as unemployment, is associated with an increased hazard ratio for disability pension at the population level [35]. Moreover, long-term unemployed people have been found to be at nearly three-fold risk of ending up with a disability pension compared to employed people, and comorbid depression has increased the risk up to 11-fold [36]. Still, as the results of the present study suggest, a lot of poor work ability among the long-term unemployed is not detected by the ordinary health services. This indicates weakness at the point of contact to the service system. The costs of care are one of the reasons for reduced visits among unemployed people [5,23], but in particular in Finland the reasons also may lie in the structures of the health services. Well-designed and comprehensive occupational health care is available, providing easily-accessible and free of charge services exclusively to the employed population, whereas the health services for unemployed people are limited to less optimally functioning municipal primary health care units and are liable to a charge [37].

In line with earlier population based studies, depression was diagnosed in health care more frequently among women, among those with the shortest unemployment period, among those who had made frequent visits to health care, and among non-manual workers [14,38,39], even the difference between non-manual workers and manual workers did not quite reach statistical significance.

Decisions on the granting of disability pensions are centralized at nation level insurance institutions in Finland. According to the Employees Pensions Act an employee is entitled to a full disability pension if her/his working ability is decreased by at least three-fifths as a result of an illness, handicap or injury, and if the work capacity cannot be restored by rehabilitation. In addition to medical reasons training, previous activities, age, and residence are taken into account as factors that relate to the ability to earn a living [30]. Such factors as the grounds for granting or rejecting the pension application are not explicated in the decisions. Concerning the present study, we could describe the processing of the application as a ‘black box’; being aware of the varying importance of depression in individual client cases. In any case, depression fulfilling the diagnostic criteria might or might not be included in the list of diagnoses in the medical certificate to support the granting of pension.

The strength of the study was the unique material, consisting of both the findings of clinical examinations and records from health care prior to the examinations. Moreover, by virtue of the EDIPE project, the group studied consisted of the hard core of long-term unemployed people that tends to be overrepresented among non-attendees and dropouts in population-based studies [14,23,40,41]. To the best of our knowledge, there is no previous research information on the identification of depression and granting of disability pensions among long-term unemployed people. Little, moreover, appears to be known about the need to assess the work ability of unemployed people.

The study also has some limitations. Because the study population had a rather long spell of unemployment and significant health and employability problems, the results cannot be generalized to the unemployed population as a whole. Diagnoses of depression, especially at the level of primary health care, may suffer from some uncertainty. As also shown in previous studies, the recording of the depression diagnosis may be missing from the records, regardless of the accurate identification [42]. We applied fairly strict criteria for depression, requiring an explicit diagnosis for a record to be valid. Furthermore, the depression diagnostics in the EDIPE examinations were not standardized in the sense of a uniform procedure. However, the diagnoses were based on clinical examination by the experienced psychiatrists engaged on the EDIPE project and adhered to the ICD-10 criteria. 

Future research should focus on the needs for treatment and assessment of working capacity among those long-term unemployed people with depression whose use of health care services is scanty and who do not actively seek treatment. To identify these people closer co-operation between employment services and health services would be essential. Furthermore, the factors closely associated with the identification of depression and granting disability pensions such as comorbidity with personality disorders, the factors connected to doctor-patient relationship, and the specific characteristics of the employment career deserve more attention in future research.

## 5. Conclusions

Screening for decreased work capacity among the long-term unemployed people appears to be required, as disability pensions are granted to two out of three people who, after undergoing thorough clinical examinations, apply for such pensions. Depression contributes to decreased work capacity in half of the applicants. Acceptance of the application is significantly more likely if depression is diagnosed in the screening examinations than when it has already been diagnosed in health care prior to the examination. Rather than the tendency of the health care professionals to ignore depressiveness in the long-term unemployed clients, the result suggests than in particular those long-term unemployed who suffer from depression are at increased risk of marginalization from health services. Novel services should be established to improve the recognition of their work disabilities and care needs among long-term unemployed people. Closer co-operation between employment and health authorities and active guidance for jobless people in using the health services especially focused on the problems connected to long-term unemployment would be crucial.

## Figures and Tables

**Table 1:** Associations of the depression (ICD-10 codes F3*) identification pattern with background variables, visits to Health Care (HC) three years preceding the EDIPE (Eligibility for a Disability Pension) project, number of somatic comorbidities, alcohol use disorder (AUD, ICD-10 codes F1*) diagnoses on the EDIPE project and granting a disability pension (DP).

	Depression Diagnosis in HC and EDIPE		Depression Diagnosis in EDIPE		No Depression		
	*n*	%	*n*	%	*n*	%	*p* ^1^
	85	23.4	103	28.3	176	48.4	
**Gender**							**0.035**
Female	41	48.2	37	35.9	56	31.8	
Male	44	51.8	66	64.1	120	68.2	
**Age**							0.662
24–39 years	6	7.1	4	3.9	13	7.4	
40–49 years	19	22.4	18	17.5	32	18.2	
50–59 years	57	67.1	72	69.9	118	67.0	
60–63 years	3	3.5	9	8.7	13	7.4	
**Marital status**							0.293
Single	57	67.1	74	71.8	134	76.1	
Married/cohabiting	28	32.9	29	28.2	42	23.9	
**Occupation**							0.050
Non-manual worker	17	20.5	16	16.2	23	13.3	
Skilled manual worker	54	65.1	74	74.7	112	64.7	
Unskilled manual worker	12	14.5	9	9.1	38	22.0	
**Duration of unemployment in quartiles**							**0.003**
1–6 years	30	35.3	24	23.3	28	15.9	
7–10 years	21	24.7	22	21.4	39	22.2	
11–13 years	11	12.9	34	33.0	53	30.1	
14–35 years	23	27.1	23	22.3	56	31.8	
**Visits to health care in quartiles (3 years prior to EDIPE)**							**0.001**
0–3	16	18.8	31	30.1	40	22.7	
4–7	16	18.8	23	22.3	48	27.3	
8–13	16	18.8	30	29.1	52	29.5	
14–72	37	43.5	19	18.4	36	20.5	
**Somatic diagnoses on EDIPE**							0.539
None	2	2.4	1	1.0	8	4.5	
1–3	42	49.4	54	52.4	88	50.0	
≥4	41	48.2	48	46.6	80	45.5	
**AUD on EDIPE**							0.775
No	42	49.4	53	51.5	95	54.0	
Yes	43	50.6	50	48.5	81	46.0	

^1^*p*-values with statistical significance are given in bold face.

**Table 2:** Associations of depression diagnosed on EDIPE (Eligibility for a Disability Pension) project and in health care (HC), on the EDIPE project only, or no diagnosed depression, with granting of the disability pension applied for.

	OR	95% CI	*p* ^1^
Model 1			
*Crude model*			
Depression diagnosis in EDIPE and in HC	1		
Depression diagnosis in EDIPE	2.16	1.18; 3.96	***0.012***
No depression	2.33	1.35; 4.00	***0.002***
Model 2			
*Adjusted for sociodemographic characteristics: Age, gender, marital status*			
Depression diagnosis in EDIPE and in HC	1		
Depression diagnosis in EDIPE	2.05	1.11; 3.79	***0.022***
No depression	2.18	1.26; 3.79	***0.006***
Model 3			
*Adjusted for: Age, gender, marital status, number of visits to HC*			
Depression diagnosis in EDIPE and in HC	1		
Depression diagnosis in EDIPE	2.35	1.24; 4.43	***0.008***
No depression	2.44	1.38; 4.31	***0.002***
Model 4			
*Adjusted for: Age, gender, marital status, number of visits to HC, number of somatic diagnoses on EDIPE*			
Depression diagnosis in EDIPE and in HC	1		
Depression diagnosis in EDIPE	2.35	1.24; 4.43	***0.008***
No depression	2.45	1.39; 4.32	***0.002***
Model 5			
*Full model. Adjusted for: Age, gender, marital status, occupational status, duration of unemployment, number of visits to HC, number of somatic diagnoses on EDIPE, AUD on EDIPE*			
Depression diagnosis in EDIPE and in HC	1		
Depression diagnosis in EDIPE	2.22	1.14; 4.33	***0.020***
No depression	2.32	1.26; 4.24	***0.007***
*Age*	*1.04*	*1.00; 1.08*	***0.045***
*Gender (male → female)*	*1.25*	*0.73; 2.13*	*0.410*
*Marital status (married → single)*	*1.42*	*0.83; 2.44*	*0.199*
*Number of visits to HC*	*1.02*	*0.99; 1.05*	*0.187*
*Number of somatic diagnoses on EDIPE*	*1.05*	*0.91; 1.21*	*0.510*
*Occupational status (skilled manual worker → non-manual worker)*	*0.38*	*0.14; 1.03*	*0.057*
*Occupational status (unskilled manual worker → non-manual worker)*	*0.41*	*0.17; 0.94*	***0.036***
*Duration of unemployment*	*1.03*	*0.98; 1.08*	*0.283*
*AUD on EDIPE*	*0.43*	*0.24; 0.70*	***0.001***

^1^*p*-values with statistical significance are given in bold face.
